# Evaluation of a Volume-Averaged Species Transport Model with Micro–Macro Coupling for Breakthrough Curve Prediction

**DOI:** 10.3390/molecules29174218

**Published:** 2024-09-05

**Authors:** Parham Mobadersani, Naine Tarun Bharat, Krishna M. Pillai

**Affiliations:** Laboratory for Flow and Transport Studies in Porous Media, University of Wisconsin-Milwaukee, Milwaukee, WI 53211, USA; mobader2@uwm.edu (P.M.); naine@uwm.edu (N.T.B.)

**Keywords:** micro–macro coupling, water filtration, porous medium, volume-averaging method, breakthrough curve, adsorption, functionalized zeolite, iron oxide, arsenic, phosphorus

## Abstract

In porous water filters, the transport and entrapment of contaminants can be modeled as a classic mass transport problem, which employs the conventional convection–dispersion equation to predict the transport of species existing in trace amounts. Using the volume-averaging method (VAM), the upscaling has revealed two possible macroscopic equations for predicting contaminant concentrations in the filters. The first equation is the classical convection–dispersion equation, which incorporates a total dispersion tensor. The second equation involves an additional transport coefficient, identified as the adsorption-induced vector. In this study, the aforementioned equations were solved in 1D for column tests using 3D unit cells. The simulated breakthrough curves (BTCs), using the proposed micro–macro-coupling-based VAM model, are compared with the direct numerical simulation (DNS) results based on BCC-type unit cells arranged one-after-another in a daisy chain manner, as well as with three previously reported experimental works, in which the functionalized zeolite and zero-valent iron fillings were used as an adsorbent to remove phosphorous and arsenic from water, respectively. The disagreement of VAM BTC predictions with DNS and experimental results reveals the need for an alternative closure formulation in VAM. Detailed investigations reveal time constraint violations in all the three cases, suggesting this as the main cause of VAM’s failure.

## 1. Introduction

The removal of non-metallic and heavy metal contaminants from water is a topic of great concern due to their non-biodegradability and toxicity [1]. Major heavy metal contaminants such as arsenic, cadmium, chromium, mercury, and lead primarily stem from various sources, including industrial processes, mining, agricultural runoff, waste disposal, urban effluents, power generation, vehicle emissions, construction, household products, and natural origins [2]. To reduce the concentration of non-metallic and heavy metals in water and wastewater to an acceptable level, several methods, such as chemical deposition, ion exchange, column coagulation, flash flotation, membrane filtration, electrochemical treatment, and adsorption, have been used [3,4]. Contaminants, such as non-metals and heavy metals, are considered environmental toxicants due to their high toxicity and persistence, and their release into the environment is caused by various natural and human activities. Arsenic is one of these heavy metals, occurring naturally in the earth’s crust and water as arsenate or arsenite. As arsenic is highly toxic, its concentration in water must not exceed 10 µg/L [3]. The removal of phosphorus plays a significant role with regard to agriculture, where rainwater runoff from fields acquires a significant loading in phosphorus due to its use in fertilizers, and which leads to the problem of algae bloom in the lakes and rivers when the runoff water falls into them [5,6,7,8]. Long-term exposure to phosphorous and arsenic in drinking water is related to various health risks, such as cancer, diabetes, heart disease, and developmental issues in children [4].

The aforementioned contaminants can be removed using the adsorption method by different adsorbents available in the market. Adsorption is extensively researched for its simplicity, affordability, straightforward operation, high processing efficiency, and effectiveness in removing many pollutants. Adsorption is a process in which a solute accumulates on the surface of an adsorbent, forming a molecular or atomic film (the adsorbate) [5]. Adsorption occurs due to the bond deficiency experienced by atoms on the surface, which are not wholly surrounded by other atoms. It is energetically favorable for them to bond with whatever is available in their immediate surroundings [6]. The selection of the adsorbent material is a critical factor in this process [9]. The most common industrial adsorbents are zeolites, activated carbon, silica gel, and iron-based materials (zero-valent iron fillings), due to their significant surface areas per unit weight [7,8,9,10].

Numerous studies have been conducted to investigate the adsorption of phosphorous and arsenic from drinking water. Researchers [6,11,12,13,14,15,16] have dedicated significant efforts to understanding the mechanisms and optimizing the adsorption process to mitigate the impact of contaminants in drinking water. The exploration of zeolite and zero-valent iron fillings (Fe(0)) as a potential method for phosphorous and arsenic removal, respectively, has attracted the attention of numerous researchers, who have extensively investigated the role of adsorbent surface area in the adsorption kinetics and removal capacities. The surface area of the adsorbent has emerged as a critical factor influencing the effectiveness of contaminant removal [6,14,15,16,17].

Several experimental explorations for the removal of contaminants such as phosphate and arsenic have been conducted [4,5,6,7,14,15,16,17,18,19,20,21,22,23,24,25]. Some of these works are briefly presented here. Bang et al. [18] investigated the efficiency of arsenic removal using zero-valent iron filings, noting that oxygen content and pH levels significantly impact the process. Arsenate removal was more efficient under oxic conditions, with an over 99.8% removal rate compared to 82.6% of arsenite at pH 6. The study highlighted the necessity of dissolved oxygen for effective arsenite oxidation and arsenic adsorption onto iron hydroxides produced by iron corrosion, emphasizing the critical role of the environmental conditions in the Fe(0) treatment process.

Leupin et al. [19] conducted a study using smaller filter columns containing iron filings and sand to treat contaminated water. The filters were designed to remove arsenic (As) and other contaminants without the need for chemical oxidants. Srivastava et al. [20] synthesized a dynamically modified iron-coated sand (DMICS) for arsenic removal, testing its efficacy in batch kinetic experiments influenced by variables like particle size, arsenic concentration, and adsorbent dosage. Particularly the column studies specify the material’s effectiveness, with significant breakthrough and exhaustion times, showcasing DMICS’s potential for arsenic removal from water [13].

Nikolaidis et al. [17] conducted two distinct field trials to evaluate the arsenic removal efficiency using iron filings. The first trial, a large-scale pilot study, operated at a flow rate of 5444 L/d to determine the BTC for arsenic. The study also included shorter column experiments focusing on system design parameters, employing smaller columns with varied dimensions and flow rates to investigate the adsorption process using zero-valent iron. These experiments aimed to derive the equilibrium-partitioning coefficient (*K_d_*) and normalize it with the adsorbent’s surface area, providing insights into the filtration system’s efficiency and the distribution of elements like sulfur within the filtration media. This comprehensive approach enabled a detailed evaluation of the arsenic filtration process, from large-scale operational feasibility to micro-scale adsorption dynamics.

In the study by Biterna et al. [22], zero-valent iron (ZVI) was evaluated for its capacity to remove arsenite from water. Batch experiments demonstrated that its removal efficiency was impacted by pH and various ions, such as Cl^−^ (chloride), CO_3_^2−^ (carbonate), and SO_4_^2−^ (sulfate), with high concentrations of borate and organic matter notably reducing the efficacy. Column tests further quantified ZVI’s performance, showing variable BTCs for different arsenic forms under flow conditions. The application of ZVI to arsenic-laden Greek groundwater revealed limitations, especially in anoxic conditions with high arsenite levels, where treated water did not meet the 10 µg/L safety guideline for arsenic. However, introducing chlorination into the ZVI treatment process significantly enhanced arsenite removal, bringing arsenic concentrations in treated water below the safety threshold.

Similarly, in an experimental study conducted by Raizada [23], analytical-grade chemicals were used to investigate zeolite’s phosphorus adsorption efficacy. A phosphorus solution, diluted to 50 mg/L, was subjected to batch adsorption tests, revealing an adsorption capacity of 5.0 ± 0.5 mg/g for zeolite. Additionally, column-flow experiments assessed zeolite’s dynamic adsorption efficiency, employing a UV–VIS Spectrophotometer to measure soluble reactive phosphorus. The findings reveal zeolite’s potential as an effective filtration material for phosphorus removal in both batch and continuous flow settings.

The above-mentioned experiments encountered challenges, including clogging and short operational lifespan. To address these problems in the case of contaminant removal, the researchers conducted a detailed laboratory study to modify the filter design, aiming to improve the removal rate while mitigating clogging and iron leaching. The modified system focused on iron corrosion preceding oxidation by dissolved oxygen, resulting in the formation of hydrous ferric oxides. The experimental study systematically evaluated parameters such as sand grain size, flow rate, and sand depth for the enhancement of filter performance.

However, the experimental works face severe challenges, such as reproducibility, cost, safety, and tedious repetitive calibrations. To overcome these hurdles, development of numerical models as a replacement of experiments have gained a lot of importance in recent years [16]. Extensive studies on adsorption processes have spurred the development of numerous models aimed at understanding and predicting adsorption behavior. These models have played an important role in understanding and optimizing the mechanisms of removal of various harmful contaminants in water through adsorption. These models serve as valuable tools for gaining insights into the adsorption mechanisms, estimating equilibrium parameters, and optimizing adsorbent materials and operating conditions. By capturing the intricacies of adsorption processes, these models can facilitate the design and implementation of effective adsorption systems for water and wastewater treatment. Among the various models proposed, the phenomenon of contaminant transport within porous media is extensively studied across various fields, such as chemical engineering, environmental engineering, soil science, and groundwater hydrology, due to its multidisciplinary relevance [26,27,28]. This complex, multiscale transport process is typically investigated through two primary length-scales used in the models: the pore scale and the lab/field scale (also called the Darcy scale). Advances in pore-scale modeling techniques and computational power have significantly contributed to the understanding and analysis of this critical issue [29,30]. Despite advancements in modeling techniques, the application of pore-scale simulations to model flows in the entire pore-space of porous media found in real world remains challenging due to the tremendous complexities associated with recreating the intricate microstructure of such pores. Any small porous filter has hundreds of thousands of pores, and each pore is unique, with very complicated, often multiscale structures. Attempting to simulate the flow and transport processes within such pores remains a nightmare despite the commonplaceness of supercomputers and parallel computers. The memory and disk space requirements remain formidable challenges even for a small cubic-inch sample of real-world porous media such as sand.

As a result, researchers have investigated this issue modeling flow and species transport in porous media by employing upscaled (upscaling is the process of coupling the small (micro) scale to large (macro) scale in porous media by using well-known, highly mathematical, and rigorous methods, such as the volume-averaging method, the homogenization method, and the mixture theory) physics leading to the development of numerous transportation models utilizing Darcy’s law in conjunction with the convection–dispersion equations.

Such transportation models based on the analytic solutions of the advection–dispersion equation have been actively carried out [31,32,33,34]. Though many transport problems are solved numerically, the analytic solutions [29,30,31,32] are still pursued by many researchers to provide better physical insights into the problems. Analytical solutions are derived from the basic physical principles and are free from numerical dispersion and other truncated errors that often occur in numerical simulations. They also provide better understanding of the contaminant process by revealing the role of parameters affecting the solute transport and adsorption process in porous media. However, these models and their analytical solutions come with inherent limitations, as illustrated in Table 1.

Alternatively, the upscaling method offers an opportunity for exploring these issues (mentioned in Table 1) by facilitating the creation of macroscale models that treat the porous medium as an averaged continuum. This technique has gained significant interest among researchers due to its proficiency in connecting pore-scale phenomena with their macroscopic analogs through the determination of effective medium coefficients of the governing equations such as Darcy’s law and the advection–dispersion equation. Upscaled models are formulated using various methodologies, such as the volume-averaging method or VAM [35,36,37], homogenization through multiple scale expansions [38,39], pore-network modeling [40], among other strategies discussed in review [39].

Pillai et al. [41] upscaled the micro-level convection–diffusion equation using the volume-averaging method to predict the evolution of contaminant concentrations in water filters. The resultant VAM equations have two possible forms, one being the classic convection–dispersion equation with its main coefficient, the total dispersion tensor, obtained from solving the PDEs associated with its closure formulation in a microstructure-based unit-cell. The other equation form includes an additional mixed derivative term with an additional vector-type transport coefficient (called the adsorption-induced vector) calculated from the two closure problems that consider the effects of passive diffusion and the adsorption of arsenic by the solid phase of the filter [41]. Such a detailed approach based on micro–macro coupling is expected to be extremely useful in practical water-filtration situations because of its potential as a reliable predictive tool with its macroscopic coefficients determined directly from the detailed microstructural features through its unique closure formulation. This is in stark contrast to the typical curve-fitting method used in the traditional advection–dispersion-equation-based modeling approaches [42,43,44,45,46,47,48,49,50].

In this paper, the VAM developed by Pillai et al. [41] was meticulously applied to three distinct experimental studies conducted by Raizada [23], Nikolaidis et al. [17], and Biterna et al. [22]. This comparison, powered by the finite-element-method-based Multiphysics solver COMSOL, aimed to rigorously test the validity and accuracy of this VAM model. The BTC obtained by VAM was tested to see if it could replicate the BTCs of three different experimental results. A DNS model was also proposed and solved to (a) check the correctness of the experiments and (b) provide a baseline result against which the VAM results could be compared. Finally, we discuss the possible reasons for the VAM failure and how to move forward.

## 2. Theory

In the theoretical section of the paper, first, the microscopic governing equations used in the direct numerical simulation (DNS) are described. Next, the equations associated with the volume-averaging method (VAM) used to upscale the microscale equations, are briefly described to establish the framework of our macroscopic equation set.

### 2.1. DNS Simulation

**Figure 1:**
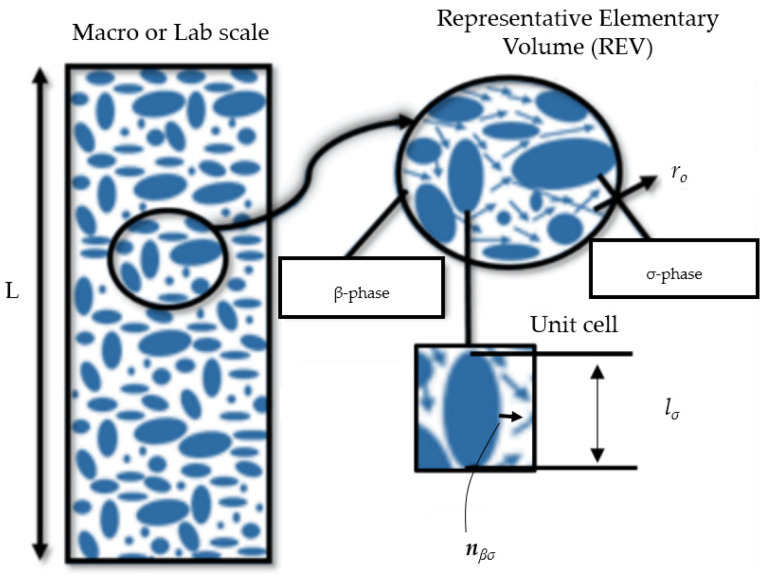
A schematic showing the relationship between the macroscopic region (where averaged equations apply) and the averaging volume (also called representative elementary volume or REV). In VAM, the REV is replaced by a unit cell after applying some assumptions. (σ-phase: solid particles; β-phase: incompressible fluid; L: column length).

DNS is a powerful computational technique that will be used to study the process of contaminant adsorption and transport in packed porous beds. DNS involves solving the governing equations for fluid flow, mass transport, and adsorption at a fine (microscopic) scale. Due to a very small Reynolds number, Stokes equation is employed to describe the fluid flow, while the advection–diffusion equation is used to model the transport and adsorption of the contaminant. By discretizing the computational domain in the pores and applying appropriate boundary conditions, DNS allows for a detailed analysis of the transport and adsorption process. Through a numerical solution of the equations, DNS provides insights into the adsorption process as well as its effect on BTCs during column tests, thus contributing to the development of effective strategies for contaminant removal. In this study, a single-phase flow occurring through a rigid and homogenous porous medium is considered (Figure 1).

As presented in Figure 1, solid particles that are rigid and stationary comprise the σ-phase, and an incompressible fluid (i.e., the contaminated water) that saturates the pores forms the β-phase. The microscale equations for flow and transport in the pore spaces and some of the important boundary conditions are presented as follows [41]:

(1) $$\frac{\partial c_{\beta }}{\partial t}+\nabla \cdot \left(c_{\beta }v_{\beta }\right)=\nabla \cdot \left(D_{\beta }\nabla c_{\beta }\right)\;$$

(2) $$B.C.1:-n_{\beta \sigma }\cdot D_{\beta }\nabla c_{\beta }=K_{eq}\frac{\partial c_{\beta }}{\partial t}\;at\;A_{\beta \sigma }$$

(3) $$-\nabla P_{\beta }+\mu \nabla^{2}v_{\beta }=0$$

(4) $$\nabla \cdot v_{\beta }=0\;$$

(5) $$B.C.2:\;v_{\beta }=0\;at\;A_{\beta \sigma }$$

In Equations (1) and (2), *c_β_* represents the concentration in the β-phase of species X (essentially representing the dissolved contaminant in contaminated water); ***v****_β_* is the velocity in the β-phase; *D_β_* equals the molecular diffusivity in the β-phase; and *K_eq_* is the equilibrium coefficient for the linear isotherm. Equation (1) can be classified as a convection–diffusion equation, where the velocity of the fluid phase is provided. The surface concentration of adsorbed ions experiences an increase due to the flux of solute ions onto the interface. It is important to note that our analysis is confined to an adsorption process that can be represented by a linear isotherm for the local mass equilibrium maintained at the *β-σ* interface. In this context, the term “*K_eq_*” represents the equilibrium coefficient (also known as the distribution coefficient) associated with the linear adsorption isotherm. By combining these two relationships, the proposed boundary condition is simplified to Equation (2).

Equation (3) is the well-known Stokes equation, which describes the fluid flow behavior under the creeping flow regime. According to Bear et al. [36], the particle Reynolds number (*Re_p_*) should be smaller than 1 for the interstitial flow to be considered as a creeping flow.

(6) $${Re}_{p}=\frac{\rho_{\beta }v_{\beta }l_{\beta }}{\mu_{\beta }}.\frac{\varepsilon_{\beta }}{1-\varepsilon_{\beta }}$$

**Figure 2:**
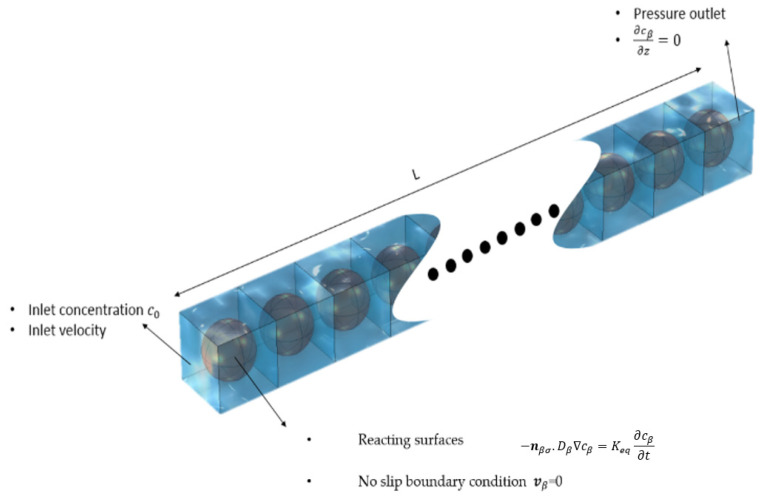
Sketch of the computational domain for the 3D system used to perform the direct numerical simulations (DNS) along with the applied boundary conditions.

Equation (4) represents the continuity equation, which ensures mass conservation at each point. Lastly, Equation (5) represents the no-slip boundary condition, specifically applied to the *β-σ* interface, which implies that the fluid velocity at the interface matches the velocity of the solid surface. Furthermore, $c_{\beta }$ at t = 0 is considered as 0; meaning that initially, there is no contaminant within the column except at the inlet wall. Figure 2 presents a 3D sketch of the computational domain along with the boundary conditions.

The packed columns with irregular solid-phase-adsorbing contaminant from water were represented in the simulation domain. The column was defined as a parallelepiped domain representing the macroscopic flow domain (i.e., the filter). It consists of a series of cubical domains linked in a daisy-chain manner. This is carried out so that the flow, species-concentration, and pressure boundary conditions at the outlet of one cubical domain are the same boundary conditions at the inlet of the down-stream cubical domain.

The cubical domain shown in Figure 2 represents the average microscopic picture in the porous water filter, within which spherical domains represent the irregular solid phase. (The size of spherical domain was chosen to match the porosity and average particle size of the filter used in the concerned experimental study). As mentioned before, Equations (3)–(5) have been used to simulate first the flow field in the cubical domains, while Equations (1) and (2) were later used to predict the solute transport and adsorption within the cubical domains in terms of local concentration fields. Appropriate boundary conditions were set at the inlet, outlet, and reacting surfaces. Initial contaminant concentrations, fluid velocity, pressure, and other variables were specified based on the concerned experimental values described in the literature. The concentration profile at the outlet is then evaluated and mapped back to the inlet boundary, where it is applied as a new inlet condition. This process is repeated, essentially solving for the concentration profile in the next (downstream) unit cell.

After the mathematical details of the DNS have been presented, the stage is set for an upscaled perspective. The focus now shifts to the VAM, introduced to establish a link between the microscopic flow and transport and the corresponding macroscopic description. Through VAM, the detailed fluid interactions identified at the particle level are upscaled, enabling their assimilation into larger-scale engineering models. As we will see below, this micro–macro coupling is marked by a powerful closure formulation. 

### 2.2. Volume-Averaging Method

The VAM is a widely used mathematical technique for upscaling and modeling a variety of problems [34] in porous media. The VAM finds extensive application in various fields, including groundwater hydrology, petroleum engineering, environmental engineering, and chemical engineering, where accurate predictions of fluid flow, heat transfer, and contaminant transport in porous media are essential [34]. In the application of VAM, in the present paper, the governing equations of fluid flow and transport are volume-averaged over a representative elementary volume (REV) within the porous medium. By averaging these equations, detailed microscale variations in flow and transport processes are smoothed out, resulting in a smoothened macroscopic representation of flow and transport in the system. This approach enables computational predictions of large-scale phenomena using macroscopic equations (such as Darcy’s law and the advection–dispersion equation for species transport) after the determination of effective properties, such as the permeability and effective diffusivity.

A brief description of VAM as applied to our problem [41] is presented here for the benefit of any uninitiated reader. In VAM, the averaging is performed over the representative elementary volume (REV) shown in Figure 1. A phase average operator ($<\varphi_{\beta }>$) and an intrinsic phase average operator ($<\varphi_{\beta }{>}^{\beta }$) are employed for upscaling, to evaluate the average value of any variable $\varphi_{\beta }$ within the entire REV or within the *β*-phase of the REV, respectively. Their mathematical definitions [34] are given as follows:

(7) $$<\varphi_{\beta }>=\frac{1}{V}\int_{V_{\beta }}\varphi_{\beta }dV\;$$

(8) $$<\varphi_{\beta }{>}^{\beta }=\frac{1}{V_{\beta }}\int_{V_{\beta }}\varphi_{\beta }dV\;$$

where the volume of the representative elementary volume (REV) and the volume of the β-phase within the REV are denoted as *V* and *V*_*β*_, respectively. These averaging operators are interconnected through the following relation:

< *φ_β_* > = *ε_β_* < *φ_β_* >*^β^* (9)

The volume fraction of the β-phase within the representative elementary volume (REV), represented as *ε*_*β*_, is closely associated with these averaging operators. Specifically, in the context of a single-phase flow of the β-phase through the porous medium, *ε*_*β*_ can be considered as the porosity of the porous medium.

By applying the averaging operators to Equations (1) to (5), certain terms emerge in the resulting point differential equations. These terms are computed by employing the spatial and temporal averaging theorems described below [34].

(10) $$<\nabla c_{\beta }>=\nabla <c_{\beta }>+\frac{1}{V}\int_{A_{\beta \sigma }}c_{\beta }n_{\beta \sigma }\;dA\;$$

(11) $$<\frac{\partial c_{\beta }}{\partial t}>=\frac{\partial <c_{\beta }>}{\partial t}-\frac{1}{V}\int_{A_{\beta \sigma }}c_{\beta }\omega \cdot n_{\beta \sigma }\;dA$$

The unit normal vector, ***n***_*βσ*_, is directed outward from the *β*-phase to the *σ*-phase (Figure 1), while the velocity of the interface between the *β*-phase and *σ*-phase is denoted as ***ω***. After the upscaling process has been conducted, the following form of the volume-averaged solute transport equation is derived in terms of the macroscopic variable, <*c*_*β*_>, i.e., the intrinsic phase average of species concentration:

(12) $$\varepsilon_{\beta }\frac{{\partial <c}_{\beta }{>}^{\beta }}{\partial t}+\nabla \cdot <c_{\beta }v_{\beta }>\;\;\;\;\;\;\;\;\;\;\;\;\;\;\;\;\;\;\;\;\;\;\;\;\;\;\;\;\;\;\;\;\;\;\;\;\;\;\;\;\;\;\;\;\;\;\;\;=\nabla \cdot \left[D_{\beta }\left(\varepsilon_{\beta }\nabla <c_{\beta }{>}^{\beta }+<c_{\beta }{>}^{\beta }\nabla \varepsilon_{\beta }+\frac{1}{V}\int_{A_{\beta \sigma }}n_{\beta \sigma }c_{\beta }\;dA\right)\right]\;\;-K_{eq}a_{\beta \sigma }\frac{\partial {{<c}_{\beta }>}_{\beta \sigma }}{\partial t}\;\;$$

The interfacial area per unit volume, *a*_*βσ*_ (calculated as $\frac{A_{\beta \sigma }}{V}$), and the average concentration over the interfacial area, ${{<c}_{\beta }>}_{\beta \sigma }$, play significant roles in this equation. The latter is defined as follows:

(13) $${{<c}_{\beta }>}_{\beta \sigma }=\frac{1}{A_{\beta \sigma }}\int_{A_{\beta \sigma }}c_{\beta }dA\;$$

To remove the pore-scale variable $c_{\beta }$ from the hydrodynamic dispersion term $<c_{\beta }v_{\beta }>$ and the area integral term on the right-hand side in Equation (12) (so that the equation can be written entirely in terms of the macroscopic variable $<c_{\beta }{>}^{\beta }$) and to transform the transient term based on ${{<c}_{\beta }>}_{\beta \sigma }$, the following standard length-scale decompositions [41] are introduced:

(14) $$c_{\beta }={<c}_{\beta }{>}^{\beta }+{\tilde{c}}_{\beta }\;$$

(15) $$v_{\beta }={<v}_{\beta }{>}^{\beta }+{\tilde{v}}_{\beta }$$

The variables ${\tilde{c}}_{\beta }$ and ${\tilde{v}}_{\beta }$ represent the spatial deviations in concentration and velocity, respectively. By substituting these pointwise defined functions and employing approximations for <*c*_*β*_>^*β*^ and <${\tilde{c}}_{\beta }$>_*βσ*_ as outlined in reference [41], a simplified version of the volume-averaged solute transport equation is obtained as follows:

(16) $$\varepsilon_{\beta }\left(1+\frac{K_{eq}a_{\beta \sigma }}{\varepsilon_{\beta }}\right)\frac{\partial {<c_{\beta }>}^{\beta }}{\partial t}+\varepsilon_{\beta }{<v_{\beta }>}^{\beta }\cdot \nabla {<c_{\beta }>}^{\beta }\;\;\;\;\;\;\;\;\;\;\;\;\;\;\;\;\;\;\;\;\;\;\;\;\;\;\;\;\;\;\;\;\;=\nabla \cdot [\varepsilon_{\beta }D_{\beta }(\nabla {<c_{\beta }>}^{\beta }+\frac{1}{V_{\beta }}\int_{A_{\beta \sigma }}n_{\beta \sigma }{\tilde{c}}_{\beta }dA)]\;\;\;\;\;-\nabla \cdot (\varepsilon_{\beta }{<{\tilde{c}}_{\beta }{\tilde{v}}_{\beta }>}^{\beta })\;$$

To estimate the unknown terms in Equation (16) involving ${\tilde{c}}_{\beta }$, Pillai et al. [41] proposed a set of equations called the closure formulation, under which a governing equation for $\tilde{c_{\beta }}$ was developed as follows:

(17) $$\frac{\partial {\tilde{c}}_{\beta }}{\partial t}+{v\;}_{\beta }\cdot \;\nabla {\tilde{c}}_{\beta }+{\tilde{v}}_{\beta }\;\cdot \;\nabla {<c_{\beta }>}^{\beta }-{\varepsilon_{\beta }\;}^{-1}\nabla \cdot <{\tilde{c}}_{\beta }{\tilde{v}}_{\beta }>\;\;\;\;\;\;\;\;\;\;\;\;\;\;=\nabla \;\cdot \;\left(D_{\beta }\nabla {\tilde{c}}_{\beta }\right)-{\varepsilon_{\beta }\;}^{-1}\nabla \cdot \;\left[D_{\beta }\left(\frac{1}{V}\int_{A_{\beta \sigma }}{n_{\beta \sigma }\tilde{c}}_{\beta }dA\;\right)\right]\;+\frac{K_{eq}a_{\beta \sigma }}{\varepsilon_{\beta }}\frac{\partial {<c_{\beta }>}^{\beta }}{\partial t}\;$$

The important boundary condition at the solid–fluid interface for Equation (17) is shown to be transformed as follows:

(18) $$-n_{\beta \sigma }\cdot D_{\beta }\nabla {\tilde{c}}_{\beta }-K_{eq}\frac{\partial {\tilde{c}}_{\beta }}{\partial t}=-n_{\beta \sigma }\cdot D_{\beta }\nabla {<c_{\beta }>}^{\beta }-K_{eq}\frac{\partial {<c_{\beta }>}^{\beta }}{\partial t}\;at\;A_{\beta \sigma }$$

The governing Equations (17) and (18), when evaluated at the closure level, were deemed quasi-steady under the following critical time-scale constraint:

(19) $$\frac{D_{\beta }\tau }{{l_{{\tilde{c}}_{\beta }\;}}^{2}}\gg 1\;$$

where τ is the characteristic time associated with changes in ${\tilde{c}}_{\beta }$ within the REV.

To solve Equation (16), the deviation in concentration, ${\tilde{c}}_{\beta }$, needs to be furnished. It was proposed that a linear combination of the source terms as

(20) $${\tilde{c}}_{\beta }=b_{\beta }\cdot \nabla {<c_{\beta }>}^{\beta }+s_{\beta }K_{eq}\frac{\partial {<c_{\beta }>}^{\beta }}{\partial t}$$

could be assumed, where ***b****_β_* (a vector field) and *s_β_* (a scalar field) are the closure variables, which are obtained from the following boundary-value problems.

Closure Problem I: (Equation set to solve ***b****_β_*)

(21a) $$v_{\beta }\cdot \nabla b_{\beta }+{\tilde{v}}_{\beta }=D_{\beta }\nabla^{2}b_{\beta \;}$$

(21b) $$B.C.1:-n_{\beta \sigma }\cdot \nabla b_{\beta }=n_{\beta \sigma }\;at\;A_{\beta \sigma }\;$$

(21c) $$Periodic\;Boundary\;Condition:\;{\;b}_{\beta }\left(r+1_{i}\right)=b_{\beta }\left(r\right),i=1,2,3,\ldots$$

(21d) $$Constraint:\;{<b_{\beta }>}^{\beta }=0\;$$

Closure Problem II: (Equation set to solve *s_β_*)

(22a) $$v_{\beta }\cdot \nabla s_{\beta }=D_{\beta }\nabla^{2}s_{\beta \;}+\frac{a_{\beta \sigma }}{\varepsilon_{\beta }}\;$$

(22b) $$B.C.1:-n_{\beta \sigma }\cdot D_{\beta }\nabla s_{\beta }=1\;at\;A_{\beta \sigma }$$

(22c) $$Periodic\;Boundary\;Condition:\;s_{\beta }\left(r+1_{i}\right)=s_{\beta }\left(r\right),\;i=1,2,3,\ldots \;$$

(22d) $$Constraint:\;{<s_{\beta }>}^{\beta }=0\;$$

Note that these problems are solved in a unit cell (shown in Figure 1b) constructed from the microstructure of any porous medium under construction. Even non-periodic media can also be represented by unit-cells because the errors associated with enforcing periodicity are not significant, provided enough number of particles are included in the unit cell [34].

This leads to the final form of the volume-averaged (macroscopic) species transport equation in terms of the upscaled concentration variable, ${<c_{\beta }>}^{\beta }$, as

(23) $$\left(1+\frac{K_{eq}a_{\beta \sigma }}{\varepsilon_{\beta }}\right)\frac{\partial {<c_{\beta }>}^{\beta }}{\partial t}+{<v_{\beta }>}^{\beta }\cdot \nabla {<c_{\beta }>}^{\beta }+K_{eq}u_{\beta }\cdot \nabla \left(\frac{\partial {<c_{\beta }>}^{\beta }}{\partial t}\right)\;=D_{\beta }^{*}:\nabla \nabla {<c_{\beta }>}^{\beta }\;$$

where the two important macroscopic coefficients used above are determined from the closure variables as follows:

(24) $$u_{\beta }={<s_{\beta }{\tilde{v}}_{\beta }>}^{\beta }-\frac{D_{\beta }}{V_{\beta }}\int_{A_{\beta \sigma }}n_{\beta \sigma }s_{\beta }dA$$

(25) $$D_{\beta }^{*}=D_{\beta }[I+\frac{1}{V_{\beta }}\int_{A_{\beta \sigma }}n_{\beta \sigma }b_{\beta }dA]-{<{\tilde{v}}_{\beta }b_{\beta }>}^{\beta }\;$$

Note that the point-wise variations in velocity inside the pores (i.e., ${\tilde{v}}_{\beta }$) are determined separately from the solution of the microscopic momentum equation, Equations (3)–(5), within the pore region. One should note that in this study, the porous medium is considered as homogeneous and isotropic. In such cases, the $D_{\beta }^{*}$ which is a 3 × 3 tensor, will be a diagonal tensor in which $D_{\beta ,xx}^{*},D_{\beta ,yy}^{*},D_{\beta ,zz}^{*}\neq 0\;$ and other terms are equal to zero [34].

In this study, Equation (23) will need to be used in its one-dimensional (1D) form along the z-direction to represent the column flow occurring in filters. Expressing the equation in its 1D form along the z-direction provides a valuable framework for studying and interpreting the system in a more concise and accurate manner.

(26) $$\left(1+\frac{K_{eq}a_{\beta \sigma }}{\varepsilon_{\beta }}\right)\frac{\partial {<c_{\beta }>}^{\beta }}{\partial t}+{<v_{\beta ,\;z}>}^{\beta }\frac{d}{dz}{<c_{\beta }>}^{\beta }{+K}_{eq}u_{\beta ,\;z}\frac{\partial }{\partial z}\left(\frac{\partial {<c_{\beta }>}^{\beta }}{\partial t}\right)\;=D_{\beta ,zz}^{*}\frac{\partial^{2}{<c_{\beta }>}^{\beta }}{\partial z^{2}}$$

**Figure 3:**
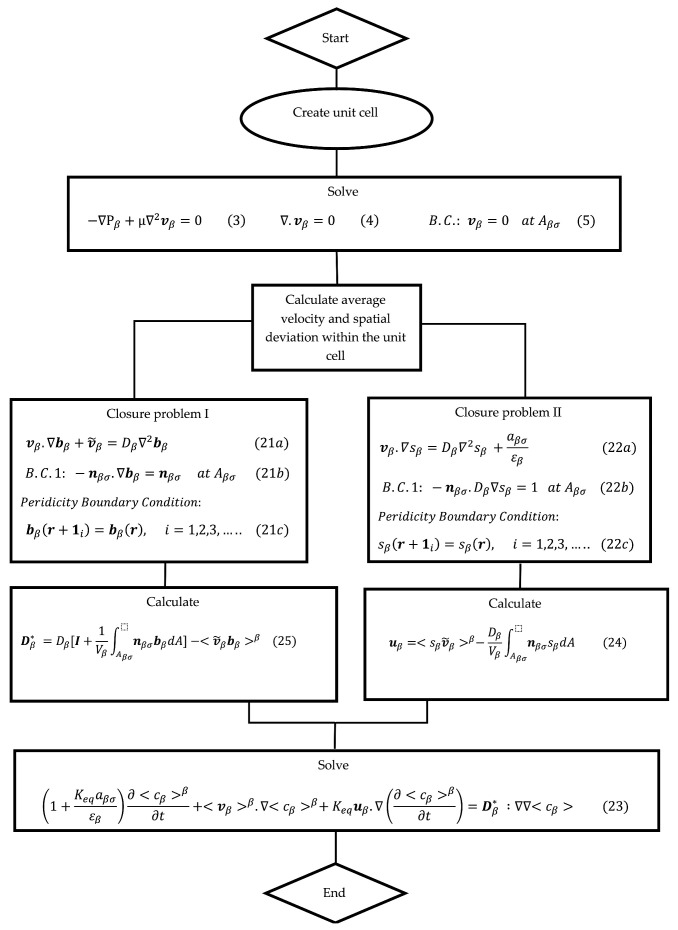
Flow chart for the VAM simulation process.

Figure 3 provides a flowchart for the VAM simulation process. It clearly presents the sequence of steps or stages involved in the VAM simulation. The first box involves solving for the Stokes flow inside a unit cell made from the microstructure of the porous filter under consideration. This allows one to determine ${\tilde{v}}_{\beta }$ field within the unit cell as well as obtain the z direction average velocity ${<v_{\beta ,\;z}>}^{\beta }$. The second box describes the equations used to solve the closure variables $b_{\beta }$ and $s_{\beta }$ within the same unit cell. Once these closure variables are integrated within the unit cell to obtain $u_{\beta }$ and $D_{\beta }^{*}$.

## 3. Numerical Simulation

In this section, to ensure the correct implementation of the VAM on COMSOL, a validation process was conducted. This involved comparing our BTC simulation results with those from a previously verified simulation [51]. By doing so, we aimed to confirm the accuracy of our COMSOL model settings and results.

### Validation of Our VAM BTC with a Result in Literature

This section presents the validation of our VAM model with respect to the significant VAM study by Valdés-Parada et al. [51], which investigates the reactive transport of a dilute substance in a Newtonian fluid flowing through a uniform porous medium under creeping flow conditions. An upscaled advection–dispersion equation was developed using VAM in [51], along with the accompanying pore-level closure. Their VAM results were validated with the help of a DNS (direct numerical simulation) involving a set of 2D unit cells with spherical particles inside.

**Figure 4:**
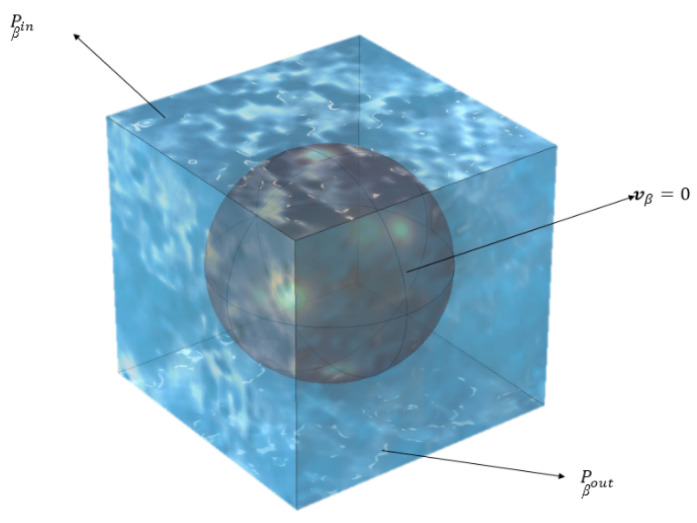
Some important boundary conditions applied to the unit cell for Stoke’s flow simulation. On the side walls, symmetry conditions were employed where the appropriate velocity gradients were set to zero.

In the computational model at the pore (micro) level, a unit cell was created such that it features a particle with a radius of 0.33 µm, positioned within a cubic cell with each side measuring 1 µm, as shown in Figure 4. The appropriate boundary conditions were implemented by us to solve Stokes’ equation within this periodic unit cell to generate the requisite pore-level velocity field. Later, the same unit cell was used to solve the closure formulation equations. Subsequent to the application of these conditions, the adsorption-induced vector, ***u****_β_*, and the total dispersion tensor, $D_{\beta }^{*}$, were determined.

**Figure 5:**
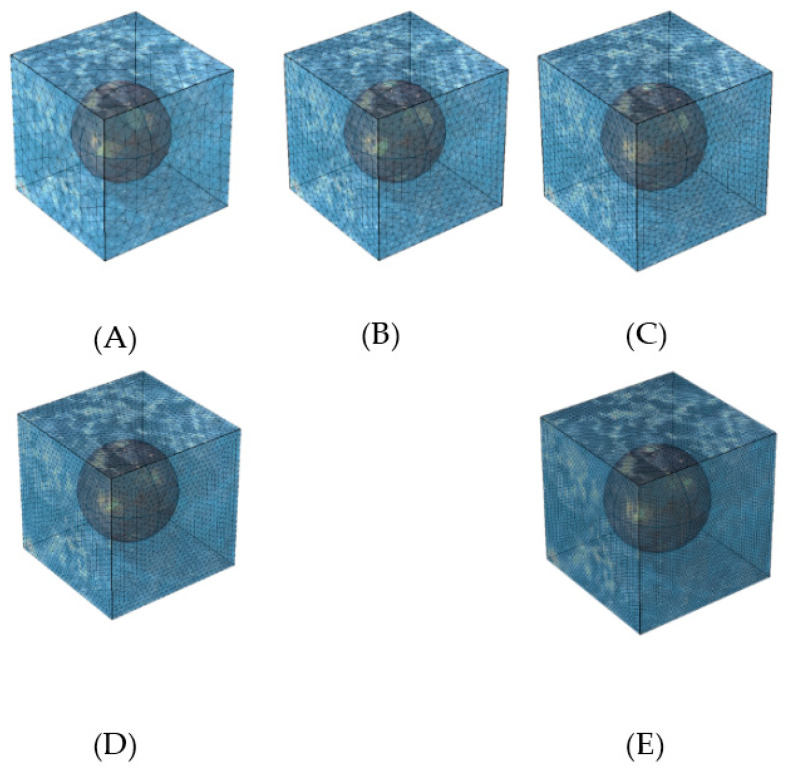
Mesh structures in unit cell studied for grid independence. (**A**) coarse, (**B**) normal, (**C**) fine, (**D**) finer, and (**E**) extra fine.

The effect of size of mesh elements in the unit cell numerical simulation is studied by utilizing five distinct levels of mesh refinement, categorized as: (A) coarse, (B) normal, (C) fine, (D) finer, and (E) extra fine. Especially, the anticipated areas with pronounced variable change and steep gradients received particular attention, with smaller element sizes assigned to such areas, in order to improve the accuracy of the model. The range of mesh densities applied in the simulations is illustrated in Figure 5.

After thorough evaluation, it was determined that the mesh D (finer mesh) yielded accurately converged results. Therefore, it was selected and utilized for numerical simulations. Additional details regarding the mesh structures can be found in Table 2.

Table 3 provides a detailed overview of the various assumptions and parameters that have been incorporated into our VAM model as well as were used by the VAM model in [51].

**Figure 6:**
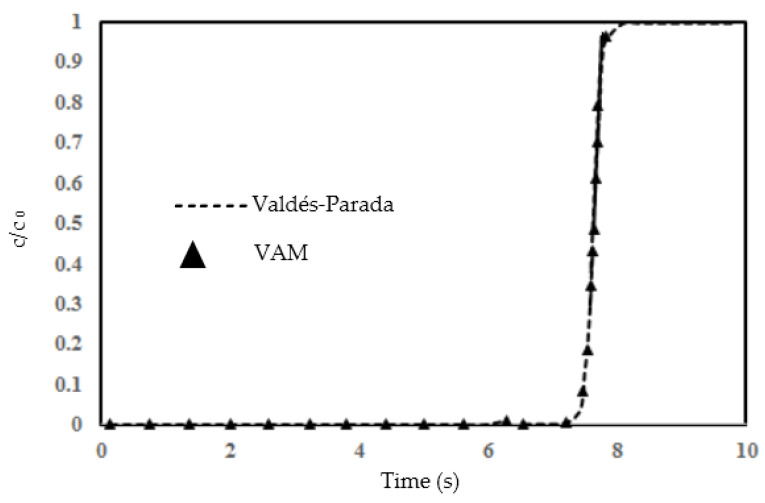
The BTC by the present VAM execution is compared with BTC predicted by VAM in [51]. The triangle symbols represent the result from the present study while the dashed line is for the work conducted in [51].

Figure 6 presents a comparison of the normalized concentration ($\frac{c}{c_{0}}$) over time at the outlet (where x = 100 mm) as predicted by the two implementations of the VAM model. This comparison shows a substantial alignment between the VAM outcomes from the present study and the VAM results obtained by Valdés-Parada et al. [51], signifying their mutual consistency in capturing the same transport phenomena within the two identical porous systems. The macroscopic simulation curve derived from our VAM implementation is in close correspondence with the curve from [51], demonstrating a remarkable overlap and thus validating our VAM implementation against the DNS-verified VAM prediction, as illustrated in Figure 6.

The graph presented in Figure 6 illustrates the validation of the current volume-averaging method (VAM) against the results obtained by Valdés-Parada [51]. The comparison is made through a BTC. The dashed line represents the findings of Valdés-Parada [51], while the triangular data points denote the outcomes from our VAM approach. From the graph, it can be observed that there is a sharp increase in normalized concentration from 0 to 1 after 7 s; this point of upsurge seems to be caused by a failure of the filtration process so that all of the contaminant is now able to pass through. The concentration then remains relatively stable at unity after the 8 s mark. Our VAM results closely follow the trend shown by Valdés-Parada’s data [51], demonstrating good agreement, particularly in the rapid increase and the subsequent plateauing. The agreement between the two studies suggests that the numerical implementation of VAM is accurate in the present study. This validation process is crucial for establishing the reliability of our VAM execution in capturing species transport in any column test, and it shows the potential applicability of the method for predictive modeling in similar scenarios. Further analysis, involving varying real time flow conditions or different chemical species, would be beneficial in confirming the versatility and accuracy of the volume-averaging method in the context of reactive transport in porous media.

## 4. Comparison of VAM BTCs with Experimental BTCs

In this study, a comprehensive comparison of the volume averaging method (VAM) results against experimental data derived from three distinct experimental studies [17,22,23] is investigated. These references have been chosen to incorporate a wide range of conditions and parameters. For clarity and structured analysis, these references are categorized into three separate cases, i.e., Case I, Case II, and Case III, each representing a unique set of experimental conditions and findings.

### 4.1. Case I: Phosphorous Removal by Zeolite [23]

Batch adsorption tests were executed to calculate the adsorption capacity of a type of functionalized zeolite. The experiment utilized a synthetic solution spiked with phosphorus, prepared using a phosphorus stock solution with a concentration of 100.0 mg/L, which was diluted with deionized (DI) water to achieve a target concentration of 50.0 mg/L. The pH of the synthetic solution was consistently recorded around 5.8 ± 0.2. 1 g of zeolite was introduced into the flask containing 200 mL of the phosphorus-spiked solution. To ensure a comprehensive evaluation of the zeolite’s capacity, a high phosphorus concentration of 50.0 mg/L was employed. The flasks were then affixed to an ORBI Benchmark Shaker plate, stirring the mixture at 180 rpm for 24 h at ambient temperature (25 ± 2 °C). After the contact period, a 2.5 mL sample was extracted from each flask to determine the residual phosphorus concentration in the solution.

**Figure 7:**
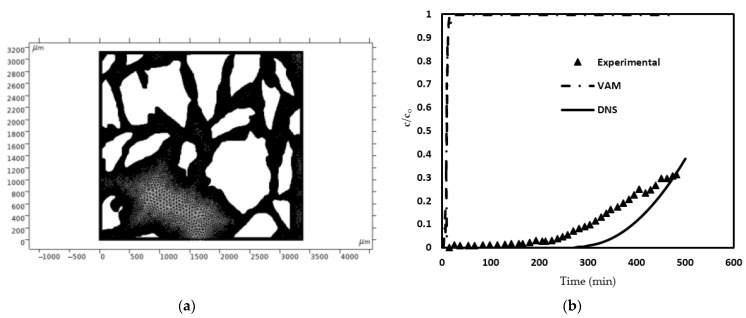
(**a**) The unit cell obtained from the micro-CT scans of zeolite-based porous filter [23]. (**b**) A comparison of the effluent phosphorous concentrations measured in the experiments and predicted using VAM [23].

The adsorptive capacity, (*q_e_*) for phosphorus on the zeolite was found to be 5.0 ± 0.5 mg/g, while the equilibrium concentration *c_e_* of the solution post-adsorption was determined to be 25.5 ± 2.3 mg/L. The adsorptive efficiency of the zeolite was measured through bench-scale column-flow experiments. An 8 g sample of functionalized zeolite granules was packed into a glass column with dimensions of 12 mm inner diameter and 275 mm length. Once the adsorbent was in place, the column material stood at a height of 85.3 mm. A peristaltic pump facilitated the flow of phosphorus-spiked solution from the influent container to the column at a consistent rate of approximately 0.02 mL/s. The plot for effluent concentration represented by the triangle symbols [Figure 7b] is set equal to the average of three experiment trials. The effluent concentration result obtained by experiments [23] is compared with the results obtained from the proposed VAM model and DNS result (shown in Figure 7b using dashed and solid line, respectively). To achieve this, our model required the microstructure details of the adsorbent (zeolite) porous bed for preparing a suitable unit cell. The micro-tomographic images of the adsorbent were captured using a micro-CT scanner, the selected domain was treated as a unit cell, and its image processing was performed using COMSOL Multiphysics; the processed image is shown in Figure 7a. Using the unit cell and the list of employed parameters (Table 4), the present VAM simulation was executed following the steps as shown in the flowchart (Figure 3). To obtain the results for effluent concentration, the Stoke’s equation [Equation (3)] relevant to the microscale, was solved within the unit cell after applying appropriate boundary conditions. At the fluid–solid interface, which represents the interface between the fluid and solid phases, the no-slip boundary condition [Equation (5)] was applied. Other b.c.s (boundary conditions), such as the symmetry b.c.s involving velocity and the pressure b.c.s at the inlet and outlet (based on experimentally observed pressure drops), were also applied. Subsequently, the computed velocity field was utilized for solving the closure problems I and II [Equations (21a)–(21d) and (22a)–(22d), respectively]. In these closure problems, the governing Equations (21a) and (22a) were solved within the pore region (or fluid domain) of the unit cell.

One should note that directly implementing the periodicity b.c.s in the original porous medium is not feasible due to the random distribution of particles. However, Whitaker [34] suggested that in such cases, the use of a periodic unit cell is admissible because the resulting errors, which are confined to the borders of the unit cell, are insignificant. This approach provides an approximation of periodic behavior within the porous medium, overcoming the challenges posed by the random particle distribution. As a result, the opposite faces of the unit cell have periodicity b.c.s [Equations (21c) and (22c)].

Lastly, the effective transfer coefficients, which capture the coupling between the micro- and macro-scale descriptions, are incorporated into the upscaled equations. These coefficients serve as key parameters that bridge the gap between the pore- and lab-scale models, allowing for an accurate representation of the system’s behavior at larger scales. The coefficients of the macroscopic Equation (23), ***u****_β_* and ***D***_β_*, are estimated by integrating through surface and volume integrals of Equations (24) and (25). The fields of the closure variables, ***b****_β_* and *s_β_*, within the pore domain of the chosen unit cell are computed from Equations (21a)–(21d) and (22a)–(22d), respectively.

On the other hand, for DNS simulation, a daisy-chain of BCC-type unit cells, containing 133 unit cells, is created in COMSOL Multiphysics representing the porous medium (Figure 2). The sphere diameter is considered as 0.72 mm, and the porosity of unit cell is 0.52. After testing the different mesh sizes and selecting the average velocity as the criteria, the finer mesh size is selected as the optimum mesh type for the DNS simulation. Finally, as shown in Figure 2, Equations (1)–(5) are solved over the porous medium.

Table 4 presents the list of parameters employed in our VAM and DNS simulations.

Figure 7b illustrates the breakthrough curve (BTC) for Case I. It is apparent that the functionalized zeolite effectively eliminated most of the phosphorus from the influent for approximately 200 min. After that, there is a gradual increase. Towards the 480 min mark, the phosphorus concentration in the effluent increased to about 15 mg/L. Furthermore, it is clear that the effluent concentration predictions made by the VAM deviated significantly from the experimental findings. The difference is very surprising since, as mentioned earlier, the results of our VAM model agreed completely with those reported in [51].

Additionally, a 3D unit cell shown in Figure 4 is created for this Case using the particle length value as the particle diameter, and then creating a cube around it such that the porosity given in Table 4 is matched. Then, our VAM model was implemented again using this altered unit cell. However, no change in the predicted BTC was observed. Hence, one may conclude that the failure of VAM is irrespective of the size, shape, and dimensionality of the unit cell.

As illustrated in Figure 7b, the VAM predictions do not align with the experimental results. A potential reason for this failure of VAM could be the violation of the time constraint mentioned in Equation (19). Consequently, we decided to evaluate the lefthand side of the time constraint, i.e., the expression $\frac{D_{\beta }\tau }{{l_{{\tilde{c}}_{\beta }\;}}^{2}}$. The characteristic time (*τ*) was calculated using two different methods, one based on just the diffusion occurring within a unit cell and the other based on both advection and diffusion occurring. (See Appendix B for more details). The results of this evaluation are presented in Table 5.

As seen in Table 5, since the value of $\frac{D_{\beta }\tau }{{l_{{\tilde{c}}_{\beta }\;}}^{2}}\;$is much smaller than unity, unlike the recommendation of Equation (19), it can be concluded that the time constraint has been violated for both diffusion and advection–diffusion approaches. This implies that the steady-state form of the closure formulation is not applicable to the present problem.

### 4.2. Case II: Arsenic Removal by Iron Filings [17]

In view of the discrepancy between VAM and experimental results (shown in Figure 7b), a similar simulation was conducted to compare our VAM model’s predictions with another set of available experimental results on the removal of arsenic [17]. Two sets of field trials were carried out by Nikolaidis et al. [17]. The first set was a large-scale, extended pilot study with a flow rate of 5444 L/d, used to assess the BTC for arsenic removal using iron filings. The second set was a series of shorter column studies examining key parameters. Shorter experiments were conducted using three smaller columns (C, D, and E) to investigate the system design parameters. They carried out batch experiments using zero-valent iron (ZVI) as the adsorbent. The experiments were conducted using conical flasks filled with 0.1 g of ZVI and 50 mL of arsenite solution with a concentration of 200 µg/L. Nikolaidis et al. [17] performed field experiments and found the *K_d_* value to be 4300 L/kg for arsenic adsorption onto iron filings or onto their surface binding. After normalization of *K_d_* with the material’s surface area, *K_eq_* is calculated to be 1.14 × 10^−4^ m.

Parameters used in our VAM simulation for this Case II study are listed in Table 6. For column C, the interfacial area per unit volume (*a_βσ_*) and porosity (*ε*) were derived from experimental details. In the study [17], the porosity given to be 0.45 was used. Iron filings typically used in heavy metal filtration studies have an effective particle size of 0.5–0.6 mm [17]. Consequently, a zero-valent iron of size 0.6 mm was assumed to approximate the size of the filings.

#### 4.2.1. DNS Simulation

For this simulation, the original filter bed consisting of ZVI particles of arbitrary shape and packed randomly in the filter bed was replaced with an ideal bed consisting of spherical ZVI particles placed at the centers of cubical unit cells in BCC lattice form, while the cells are arranged regularly in square arrangement in space. The sphere diameter was the same as the particle diameter, while the size of the unit cell was decided by the porosity of the filter bed. (See Table 6 for the values of these parameters). In this case, 754 unit cells formed the studied porous medium.

**Figure 8:**
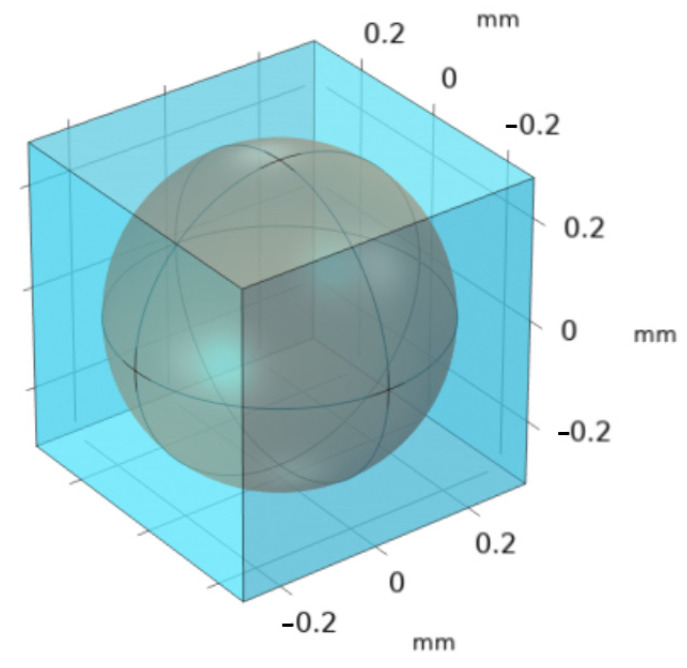
The structure of the unit cell for solving the closure formulation. The fluid domain is shown in light blue color while the solid phase in the form of a spherical particle is shown in brown.

A numerical simulation of the flow and transport in unit cells accompanied by adsorption of arsenic on spherical ZVI particles was conducted using COMSOL Multiphysics. The column was defined as a cubical domain shown in Figure 8. These unit cells were assumed to be arranged in a daisy-chain fashion (i.e., one behind the other) within the column. A mesh was generated on this domain with a fine enough resolution to accurately capture the physical processes under consideration. As mentioned before, Equations (3)–(5) were solved to simulate the arsenic adsorption within the column during Stoke’s flow. Appropriate boundary conditions were set at the inlet, outlet, and walls of the column. Initial concentrations of arsenic, fluid velocity, and other parameters listed Table 6 were used for computing the concentration distributions in the domain.

#### 4.2.2. VAM Simulation

COMSOL Multiphysics software is employed to discretize the computational domain, specifically the pore region within the unit cell (shown schematically in Figure 1b), using triangular elements. This discretization process facilitates the numerical analysis of fluid flow within the porous medium and enables the accurate modeling and simulation of the system using COMSOL’s computational tools.

In this particular investigation, a body-centered cubic (BCC)-type unit cell was constructed (shown in Figure 8), featuring a central sphere of solid phase positioned within its confines. This unit cell is a microstructural representative of the porous filter bed used in [17] and served as the basis for this study. Employing the periodic boundary conditions, the closure problems I [Equation (21a)–(21d)] and II [Equation (22a)–(22d)] were solved. The velocity distribution for the unit cell was estimated by solving Equations (3) and (4), and the no-slip boundary condition, Equation (5). To determine the pressure-drop along the length of the unit cell, Darcy’s law and the Kozeny–Carman equation for permeability were used. (See Appendix A).

To assess the impact of different mesh sizes on the VAM solutions, the model was discretized as before using five distinct mesh configurations, namely (A) coarse, (B) normal, (C) fine, (D) finer, and (E) extra fine. Moreover, to achieve higher accuracy in areas where changes in variables are severe and large gradients are anticipated, the cell size was made smaller. Additional details regarding these meshes can be found in Table 7.

**Figure 9:**
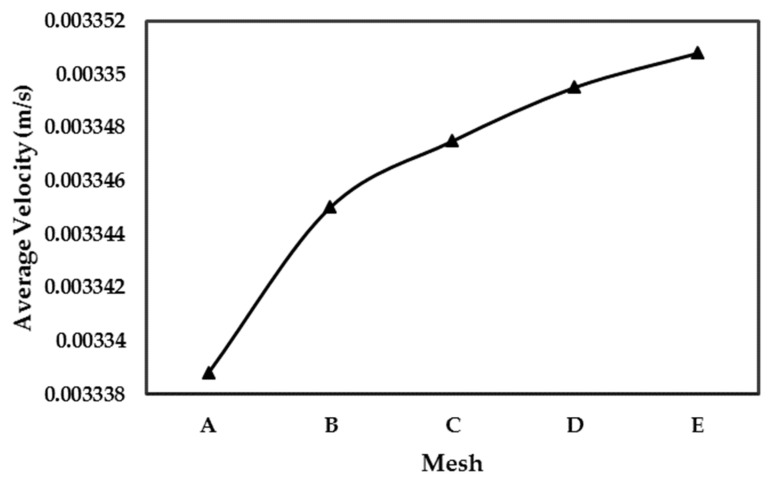
Grid independence study for Case II.

Figure 9 shows the comparison of average velocity obtained for different mesh considered. After thorough evaluation, it was determined that the mesh D (finer mesh) yielded converged results. Therefore, it was selected and utilized for numerical simulations. Applied boundary conditions are presented in Figure 4.

**Figure 10:**
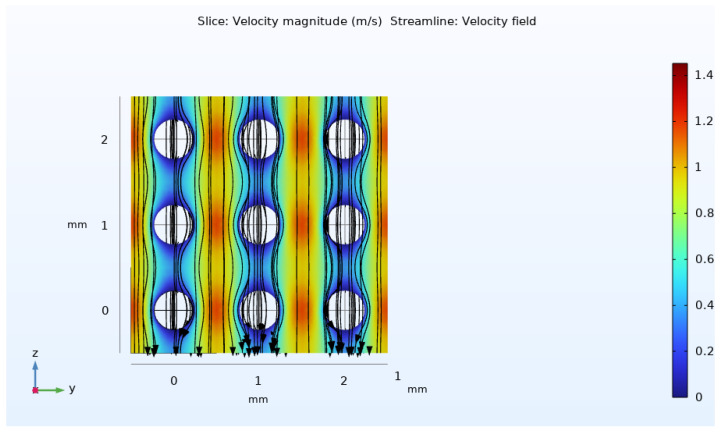
Streamline and velocity distribution in a few unit cells arranged in daisy-chain manner behind one another.

By the way, under the given experimental data of [17], the particle Reynolds number (Equation (6)) is calculated as 0.36, which satisfies the creeping flow criteria. Therefore, the use of the Stoke’s equation for determining the velocity field is justified. As presented in Figure 10, a semi-symmetric velocity distribution is obtained at the pore level in this simulation. Due to the applied pressure difference, the fluid flows from top to bottom. Maximum velocity occurs in the narrow gaps around the spherical solid phase. Based on this simulation, the average velocity within the unit cell is estimated to be about 3 mm/s using Equation (8).

**Figure 11:**
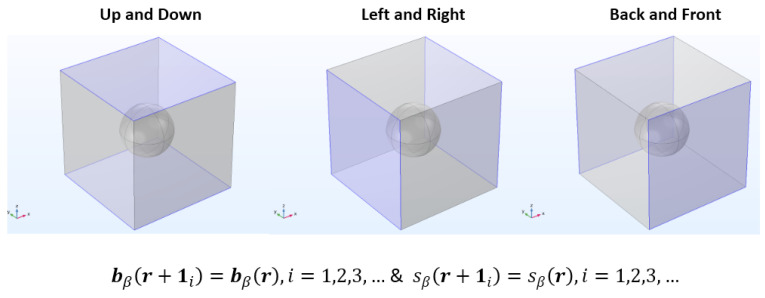
Schematic representation of the three sets of faces of the unit cell where the periodic boundary conditions of the closure formulation, Equations (21c) and (22c), are applied.

Subsequently, the calculated velocity field is utilized to solve both the closure problems of I and II type (Equations (21a)–(21d) and (22a)–(22d)). The governing differential equations are solved within the pore area or fluid domain of the unit cell. Consequently, the periodicity boundary conditions (Equations (21c) and (22c)) are applied to the opposing borders of the periodic unit cell, as shown in Figure 11.

**Figure 12:**
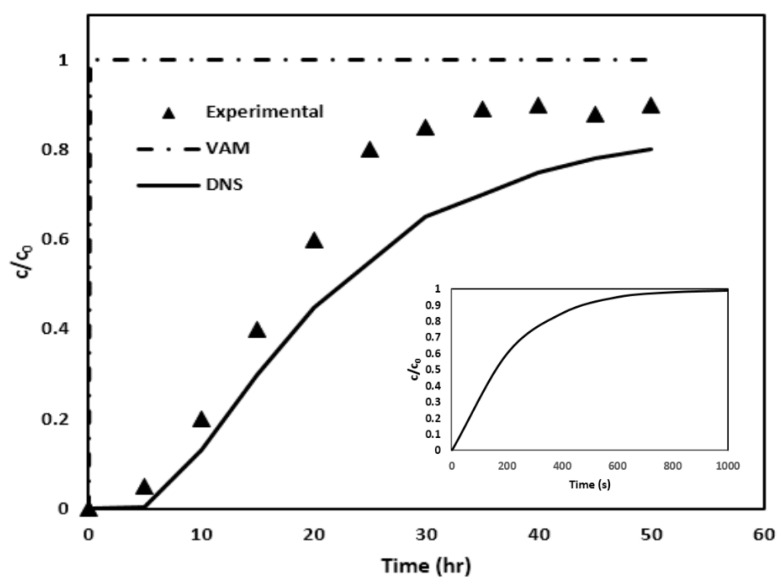
The BTCs predicted by VAM, DNS, and the experiments are compared for Case II. The inserted figure shows the plot at an enlarged time scale for the VAM result.

After simulating the creeping flow and consequently calculating the ${\tilde{v}}_{\beta }$ by the use of Equation (15), the two sets of closure formulation PDEs are solved to determine the distributions of the closure variables, ***b_β_*** and *s_β_*, within the pore region. The closure variables ***b****_β_* and *s_β_* are then utilized in Equations (24) and (25) for evaluating important macroscopic coefficients ***u****_β_* and $D_{\beta }^{*}$, respectively, through surface and volume integrals. Equation (26) is then solved to determine the evolution of the average arsenic concentration during the column flow. The resultant BTC, shown in Figure 12 with the help of long-dash line, is then finally obtained.

In order to check the accuracy of the developed VAM model, the arsenic BTC from VAM simulation is compared with experimental results obtained by Nikolaidis et al. [17], as shown in Figure 12. As a reference, the DNS results are also included. It is apparent that the VAM BTC is way off the mark. In comparison, the DNS BTC is closer. Once again, as in Case I, the VAM appears to be failing miserably.

In exploring the discrepancy between the experimental results and the predictions made by VAM, one single contributory factor has been identified. This discrepancy may stem from a violation of the time constraint discussed in Equation (19). The result is that we are solving a steady-state type of closure problem instead of a transient one, which seems to be seriously affecting accuracy of BTC prediction.

Similar to Case I, the characteristic time used in Equation (19) was calculated by solving the pure diffusion equation on the one hand, and the advection–diffusion equation on the other. The results of these calculations are presented in Table 8. (See Appendix B for details).

As in Case I, the time constraint has also not been satisfied for Case II. This conclusion is evident from the data presented in Table 8, which shows that both the diffusion and advection–diffusion approaches fail to meet the required time constraints for this case.

### 4.3. Case III: Arsenic Removal by Iron Filings [22]

As the third reference, the batch and column tests conducted by Biterna et al. [22] have been selected for testing the accuracy of BTC predicted by VAM. Their study also investigates the efficacy of zero-valent iron (ZVI) in the removal of arsenite, a toxic form of arsenic, from water sources.

A series of batch experiments were designed and executed. These experiments aimed to evaluate how different pH levels influence the effectiveness of ZVI in the removal of arsenite. In the experimental setup, Erlenmeyer flasks were prepared, each containing a precise amount of 0.1 g of zero-valent iron (ZVI). To these flasks, 50 mL of an arsenite solution with a concentration of 200 µg/L was added. The initial pH of each mixture was adjusted to 7.0 ± 0.2 using either 0.1 M hydrochloric acid (HCl) or sodium hydroxide (NaOH), ensuring a consistent starting point for all tests. All these experiments and controls were conducted under controlled room temperature conditions, maintained at approximately 23 ± 1 °C, to eliminate temperature variations as a variable in the study.

Columns made of Pyrex glass, each with an inner diameter of 1.2 cm and lined with glass wool at the base, were prepared and filled with fine sand particles with the average diameter of 0.45 mm, and 1 g of zero-valent iron (ZVI). The total height of the packed bed in each column was maintained at 10 cm, with a porosity calculated to be 0.62. To test the columns, tap water was spiked with arsenite, ensuring a final concentration of 100 µg/L. This contaminated water was then made to flow through the columns in a downward direction, forced by a peristaltic pump at a rate of 1 L/hour. The pH of this influent water was carefully controlled to be 7.5 ± 0.2. At specific time intervals, samples of the effluent water were collected for analysis. In these samples, concentrations of As and ferrous iron [Fe(II)], the oxidation-reduction potential (Eh), and pH were measured to assess the treatment efficacy. Additionally, the performance of the ZVI column was tested using natural groundwater samples, labeled A and B, which had high levels of arsenic and varied in their physicochemical properties. Table 9 provides all the required parameters for numerical simulations obtained from [22].

**Figure 13:**
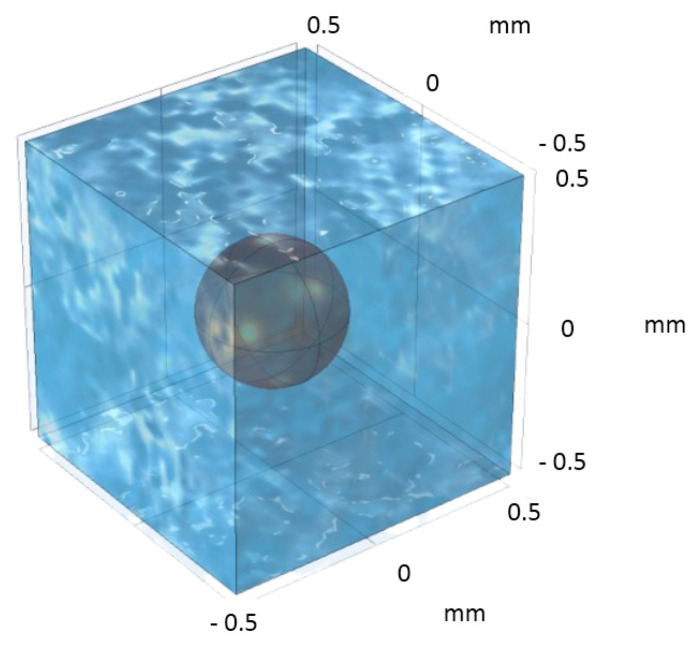
BCC-type unit cell with a central solid-phase (ZVI) particle of spherical shape located at the center of a cube.

Applying the same approach as outlined in the second reference, a BCC-type unit cell was constructed for the analysis of Case III. For this unit cell, the diameter of the spherical ZVI particle was assumed at 0.45 mm. Correspondingly, the unit cell shown in Figure 13 was designed, positioning the ZVI particle centrally within the cubic cell. This configuration facilitates a focused examination of the particle’s interaction within its immediate environment, ensuring that the analysis remains consistent with the methodologies employed in the initial reference. It is important to note that for this DNS simulation, the daisy chain consisted of 196 unit cells.

To evaluate the effects of various mesh configurations on the outcomes, the model tested five levels of mesh refinement: (A) coarse, (B) normal, (C) fine, (D) finer, and (E) extra fine. Additionally, regions experiencing significant variable–value changes and substantial gradients were modeled with reduced finite element sizes to enhance accuracy. Detailed information about the tested mesh configurations is provided in Table 10.

**Figure 14:**
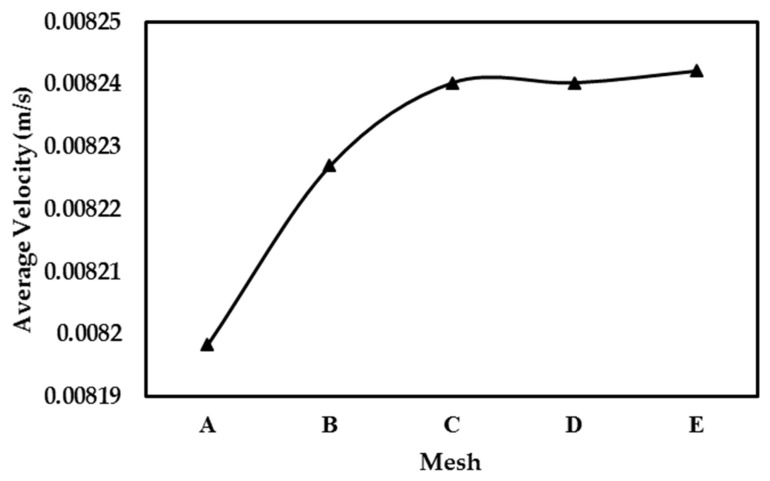
Result of grid-independence study for Case III.

Similar to the earlier two cases (Case I and Case II), the grid independence study for Case III using different meshes is shown in Figure 14. Here, changes in the average velocity, estimated by dividing total flow-rate with the unit-cell cross-section area, with increasing mesh density are studied. Following this evaluation, mesh C (fine mesh) was found to produce results with accurate convergence. Consequently, this mesh was chosen for use in the subsequent numerical simulations. Finally, the steps shown in flowchart of Figure 3 are implemented to conduct the VAM simulation and to obtain the BTC for the present case, Case III.

**Figure 15:**
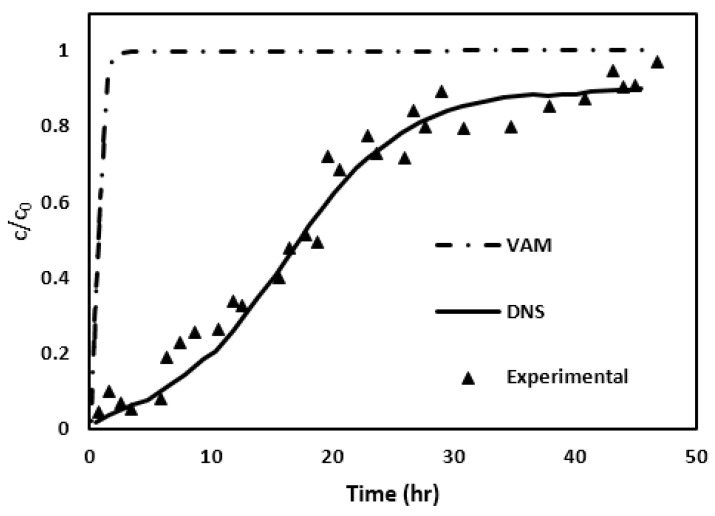
VAM, DNS, and experimental result comparison. The inserted figure shows the enlarged time scale for the present VAM.

In Figure 15, the break-through curves (or BTCs) obtained through three different means are shown; it is a comparative analysis between DNS, VAM, and experimental data [22]. The DNS BTC, represented by a solid line, demonstrate a gradual increase in normalized concentration over the observed duration. This incremental trend provides an excellent match with the trend seen in the column experiments. In contrast, the VAM BTC, indicated by a dotted line, is characterized by a sharp, step-like rise to the higher normalized concentration. Similar to the Cases I and II, Figure 15 clearly demonstrates that the VAM BTC prediction in Case III also fails spectacularly when compared with the experimental findings. Once again, this inconsistency could perhaps be attributed to the failure of the time constraint outlined in Equation (19). To investigate this issue, the characteristic time used in Equation (19) was computed by solving the diffusion and advection–diffusion equations in the unit cell geometry as described in Appendix B. Table 11 lists the values of the characteristic time and the left-hand side of the time constraint [Equation (19)].

Similar to Cases I and II, the time constraint has not been satisfied for Case III since $\frac{D_{\beta }\tau }{{l_{{\tilde{c}}_{\beta }\;}}^{2}}$ values are much smaller than unity. This conclusion is supported by the data in Table 11, where calculations for both the diffusion and advection–diffusion approaches demonstrate a failure to meet the required time constraint.

## 5. Discussion

The main aim of this study was to authenticate the effectiveness of the volume-averaged method (VAM) for predicting the breakthrough curves (BTCs) seen in column tests for filtration of phosphorus and arsenic. In this method, the VAM-based upscaled convection–diffusion equation is used that ensures micro–macro coupling using the closure formulation. The graphical results generated for three distinct cases (Cases I, II and III) provide a side-by-side evaluation of the VAM predictions against the DNS predictions and the experimental findings. It is understood from Figure 7b, Figure 12 and Figure 15 that the VAM does not align closely with the experimental data in the initial and middle phases of the adsorption process, emphasizing its failure in simulating the experimentally observed BTCs. (Surprisingly, the DNC BTC performs better as it has a much better match with the experimental BTC.) This indicates that there is something fundamentally wrong with the VAM currently proposed, with its emphasis on steady-state type closure formulations [41].

In this study, as a possible reason for the VAM failure, the time constraint, as presented in Equation (19), was assessed for all the three experimental studies, with the findings presented in Table 12. (See Appendix B for estimating the characteristic times).

As presented in Table 12, in all three examined cases (Case I, Case II, Case III), since the left-hand side was much lower than 1, the time constraint was violated. This was observed whether a pure diffusive approach was used to estimate the time constant or a more realistic approach of solving the full advection–diffusion equation within the unit cell. We feel that the time-constraint violation is the most important reason for the inaccurate prediction of BTC by VAM. This matches the sentiments expressed by Whitaker (page 142, [34]), that the time-constraint “is not always satisfied for typical laboratory experiments, and unsteady closure problems may be necessary for the interpretation of some laboratory experiments”.

(One can of course proffer other explanations for the inaccurate predictions by VAM. Additional variables, including the lack of periodicity in real porous media, inherent randomness of porous filters, uncertainty in property values, simplification inherent in the 1D equation approach (i.e., ignoring 3D effects), etc., could be marshaled to explain the discrepancies observed with VAM predictions. However, these reasons are in the realm of speculation as they were not tested in a systematic manner in the present study).

## 6. Conclusions and Future Work

In this paper, our primary objective was to evaluate the VAM (volume-averaged method), a rigorous mathematical technique that relies on the PDE-based closure formulation to ensure micro–macro coupling during upscaling from micro (pore) scale to macro (lab) scale, in predicting the contaminant transport and adsorption in porous water filters for removing phosphorus and arsenic. The implementation of the VAM was validated by testing the simulated BTCs (breakthrough curves) in column tests against real experimental results. In this study, three different experimental results from the literature were selected as references for the evaluation of the VAM. A DNS based on a BCC-type unit cell with a solid phase as a sphere at the center, and the unit cells set one after another in daisy chain manner, was also tested. Surprisingly, the BTCs predicted by this DNS were found to be much closer to the experimental BTCs. Comparing the experimental BTC with the simulated VAM results, it was shown that there is a serious disagreement between VAM BTCs, DNS BTCs, and experimental findings. Investigating the reason behind these failures, the time constraints assumed during the derivation of the closure formulation were studied for all the three cases (Case I, Case II, Case III) for both pure diffusive and complete advective–diffusive transport scenarios. In all cases, the time constraint was violated significantly.

However, the need for a successful, validated VAM is important for developing an upscaling theory with micro–macro coupling. Therefore, as a future prospective, we invite VAM workers to propose a new closure formulation that follows the time constraint in the context of the mentioned practical water-filtration applications. As suggested by Davit and Quintard [52], the new closure form will involve integrodifferential equations with temporal convolutions. The other possible solution is adoption of some type of hybrid VAM model where adsorption into the solid phase is simplified without using any type of detailed closure formulation. The transient-closure approach proposed by Valdes-Parada and Sanchez-Vargas [53] also suggests a way forward. However, there are a few problems with it while comparing with actual water filtration experiments. In the suggested paper, the authors compare their general upscaled model with a version of their quasi-steady model for different Peclet numbers. Figure 7 in that paper shows that in the low Peclet number regimes (corresponding to the low Reynolds number of ours), the two predictions match very well. We suspect that the adoption of the transient closure suggested in this paper will yield a profile that is very close to the one obtained by us. Also, in [53], the predictions were never compared with real-life experimental cases. But in any case, it provides a new theoretical direction, and it is worth studying to see if a similar approach can be developed for the water filtration application outlined here.

The payout of this future work for water filtration research is expected to be significant. The new validated VAM will allow the engineers and researchers to ‘design’ a porous filter in terms of its pore-level microstructure, which will be characterized in terms of measures such as porosity, pore size distribution, average particle size, particle size distribution, anisotropy, etc. The resulting VAM theory and simulation will also be the ‘contaminant agnostic’, i.e., they can be easily adapted for the removal of any known contaminant, such as arsenic, lead, cadmium, phosphorus, etc., as well as the newly emerging contaminants such as PFAS.

## Figures and Tables

**Table 1:** Limitations and effects of analytical models.

Limitations	Effects
**Assumptions on System Homogeneity and Isotropy** [35]	While facilitating the mathematical tractability, these simplified assumptions can deviate significantly from the complex heterogeneous character of natural systems, potentially compromising the prediction accuracy.
**Uncertainties in Parameter Estimation** [36]	The inherent variability and uncertainty in these parameters (such as the dispersion coefficient and hydraulic conductivity) across real-world environments can significantly undermine the reliability of model outputs.
**Exclusion of Complex Interactions** [37]	Ignoring the critical physical, chemical, and biological interactions (including microbial activity, plant uptake, and phase changes) that influence solute transport leads to oversimplified representations of the solute dynamics.
**Idealized Boundary and Initial Conditions** [38]	Predefined idealized boundary and initial conditions, such as infinite domains or constant source concentrations, rarely align with the complexities observed in natural and engineered systems, affecting the applicability of the models’ predictions.

**Table 2:** Details of different meshes tested on the unit cell.

Mesh	Triangles	Element	Vertex
A	1904	10,304	2840
B	4114	29,262	7293
C	6536	57,512	13,397
D	12,956	159,039	34,010
E	33,664	553,344	111,574

**Table 3:** Parameters employed in the numerical validation study of the VAM advection–dispersion equation and the associated closure problems.

Parameter	Description	Value	Unit
L	Column height	100	mm
$\varepsilon_{\beta }$	Porosity	0.85	–
$a_{\beta \sigma }$	Interfacial area per unit volume	1600	1/m
$D_{\beta }$	Molecular diffusivity	10^−9^	m^2^/s
$c_{0}$	Initial concentration	100	µg/L
$v_{\beta }$	Pore velocity	0.003	mm/s
$d_{\sigma }$	Particle diameter	0.66	mm

**Table 4:** Parameters employed in the numerical simulations of closure problems of Case I.

Parameter	Description	Value	Unit
L	Column height	100	mm
$\varepsilon_{\beta }$	Porosity	0.52	–
$a_{\beta \sigma }$	Interfacial area per unit volume	3928	1/m
$D_{\beta }$	Molecular diffusivity	6.2 × 10^−10^	m^2^/s
$c_{0}$	Initial concentration	50	mg/L
$v_{\beta }$	Pore velocity	0.36	mm/s
$l_{\sigma }$	Particle length	0.72	mm

**Table 5:** Time constraint calculation for Case I. Two types of physics were considered: one of pure diffusion and the other of advection and diffusion. See Appendix B for details.

Diffusion		Advection-Diffusion	
*τ*	$\frac{D_{\beta }\tau }{{l_{{\tilde{c}}_{\beta }\;}}^{2}}$	*τ*	$\frac{D_{\beta }\tau }{{l_{{\tilde{c}}_{\beta }\;}}^{2}}$
68.7 s	0.076	53 s	0.059

**Table 6:** List of parameters used in VAM simulation for Case II (taken from Column C experimental data of [17]).

Parameter	Description	Value	Unit
L	Column height	46	cm
d	Column diameter	10	cm
$\varepsilon_{\beta }$	Porosity	0.45	–
$a_{\beta \sigma }$	Interfacial area per unit volume	375	1/m
$D_{\beta }$	Molecular diffusivity	11.6 × 10^−10^	m^2^/s
$c_{0}$	Initial concentration	200	µg/L
$v_{\beta }$	Pore velocity	1.07	mm/s
$d_{\sigma }$	Particle diameter	0.6	mm

**Table 7:** Different meshes applied to the Case II unit cell in a grid independence study for the VAM solution.

Mesh	Triangles	Element	Vertex
A	7712	9080	2459
B	22,507	25,365	6147
C	45,531	50,103	11,380
D	128,483	137,581	28,874
E	438,176	461,730	91,793

**Table 8:** Time constraint calculation [i.e., the value of the left-hand side of Equation (19)] is performed for Case II using the time scales estimated using the pure diffusion physics as well as the full advection–diffusion physics. (See Appendix B for details).

Diffusion		Advection-Diffusion	
*τ*	$\frac{D_{\beta }\tau }{{l_{{\tilde{c}}_{\beta }\;}}^{2}}$	*τ*	$\frac{D_{\beta }\tau }{{l_{{\tilde{c}}_{\beta }\;}}^{2}}$
22 s	0.068	17.1 s	0.053

**Table 9:** A compilation of the parameter values sourced from the data on column tests conducted in reference [22].

Parameter	Description	Value	Unit
L	Column height	10	cm
d	Column diameter	1.2	cm
$\varepsilon_{\beta }$	Porosity	0.62	–
$a_{\beta \sigma }$	Interfacial area per unit volume	375	1/m
$D_{\beta }$	Molecular diffusivity	10^−9^	m^2^/s
$c_{0}$	Initial concentration	100	µg/L
$v_{\beta }$	Pore velocity	0.66	mm/s
$d_{\sigma }$	Particle diameter	0.45	mm

**Table 10:** Details of five different FEM meshes used to conduct mesh refinement study in the pore region of the unit cell used in Case III.

Mesh	Triangles	Element	Vertex
A	7786	10,628	3630
B	19,548	24,748	7518
C	34,964	43,256	12,581
D	108,190	125,028	31,647
E	395,426	440,272	101,638

**Table 11:** Time-constraint-related calculations [Equation (19)] for case III.

Diffusion		Advection–Diffusion	
*τ*	$\frac{D_{\beta }\tau }{{l_{{\tilde{c}}_{\beta }\;}}^{2}}$	*τ*	$\frac{D_{\beta }\tau }{{l_{{\tilde{c}}_{\beta }\;}}^{2}}$
16.7 s	0.064	9.4 s	0.036

**Table 12:** Evaluation of the left hand side of the time constraint [Equation (19)] for all the three experimental cases (Case I, Case II, Case III).

Case Number	Particle Diameter	Characteristic Time	$\frac{D_{\beta }\tau }{{l_{{\tilde{c}}_{\beta }}}^{2}}$ (Pure Diffusion)	$\frac{D_{\beta }\tau }{{l_{{\tilde{c}}_{\beta }}}^{2}}$ (Advection-Diffusion)
Case I	0.72 mm	68.7 s	0.076	0.059
Case II	0.6 mm	22 s	0.068	0.053
Case III	0.45 mm	16.7 s	0.064	0.036

**Figure A1:**
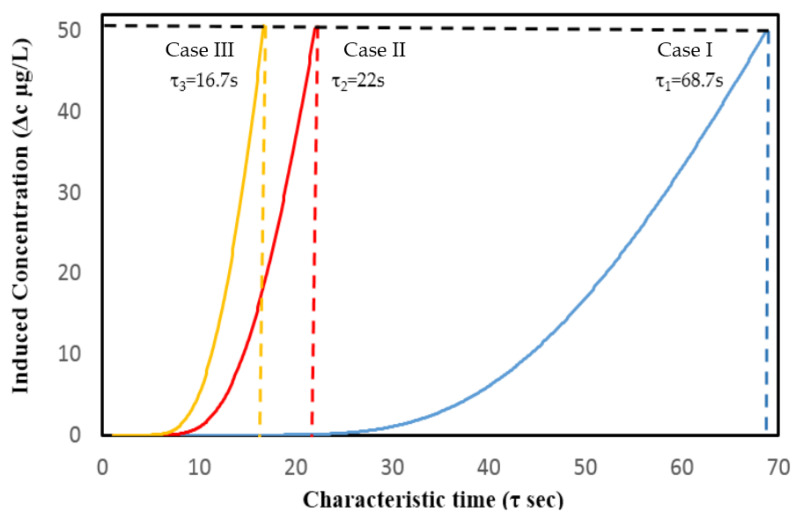
Comparison plot for the induced concentration and characteristic time (τ), represented by dashed color line, for Cases I, II, and III.

**Figure A2:**
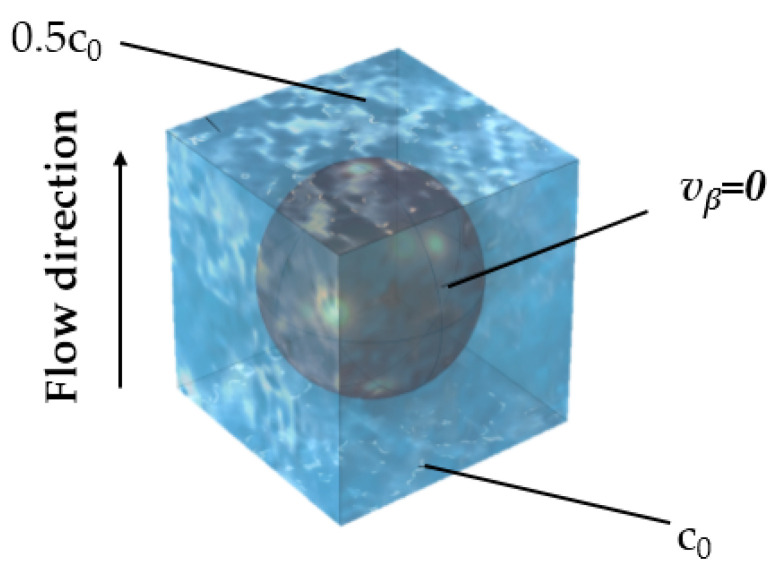
Boundary conditions for the CFD simulation conducted for estimating the characteristic time.

**Figure A3:**
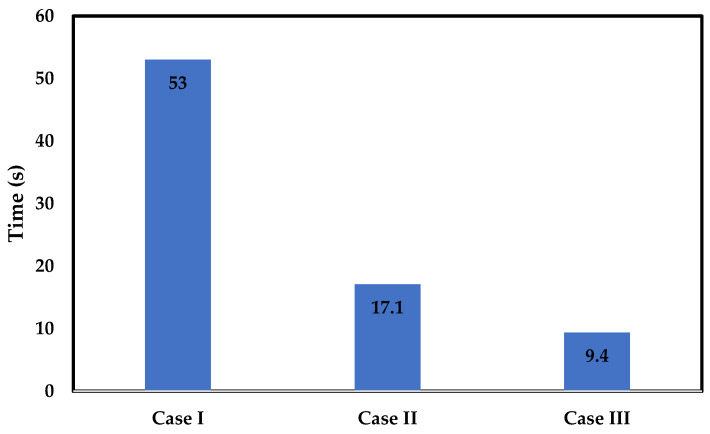
Characteristic time for Cases I, II, and III estimated by solving the species transport convection–diffusion equation within the unit cell.

## Data Availability

Data are contained within the article.

## Nomenclature

L	Characteristic length associated with the macroscale	m
d	Characteristic diameter associated with the macroscale	m
*c_β_*	Point concentration of species X in the β phase	mol/m^3^
***v*** *_β_*	Velocity of the β phase	m/s
*D_β_, D*	Molecular diffusivity in the β-phase	m^2^/s
***n*** *_βσ_*	Unit normal directed from β phase to σ phase	
*K_eq_*	Equilibrium coefficient for the linear isotherm.	m
*A_βσ_*	Interfacial area between the β and σ phases in the REV	m^2^
*ε_β_*	Porosity	
*a_βσ_*	The βσ interfacial area per unit volume	1/m
< >^β^ (Operator)	Intrinsic phase average	
< > (Operator)	Phase average	
*V_β_*	Volume of the β phase in REV	m^3^
${\tilde{c}}_{\beta }$	Spatial deviation in concentration of the β phase	mol/m^3^
${\tilde{v}}_{\beta }$	Spatial deviation in velocity of the β phase	m/s
***b*** *_β_*	Closure variable (vector) used to describe the distribution of ${\tilde{c}}_{\beta }$	m
*s_β_*	Closure variable (scalar) used to describe the distribution of ${\tilde{c}}_{\beta }$	s/m
***u*** *_β_*	Adsorption-induced vector in the β phase	
***D*** *^*^* *_β_*	Total dispersion tensor	m^2^/s
τ	Characteristic time	s
P_β_	Hydrodynamic fluid pressure in the β-phase	Pa
V	Volume of the REV	m^3^
$\varphi_{\beta }$	Generic variable of the β-phase	
$l_{\tilde{c_{\beta }}}$	Characteristic length-scale for variation in ${\tilde{c}}_{\beta }$	m
C_0_	Reference concentration used in the analytical solution for a finite Domain	mol/m^3^
*d_σ_*	Particle diameter	m
*Q*	Flow rate	m^3^/s
*Re_p_*	Particle Reynolds number	
*ρ_β_*	Fluid density	kg/m^3^
*µ_β_*	Fluid Dynamic viscosity	N.s/m^2^
*k*	Permeability of the porous medium	m^2^
$l_{\beta }$	Length of the *β* phase in REV	m
*M*	Amount of detectable tracer concentration per unit area	mol/m^2^

## Appendix A. Calculation of Pressure Drop over a Unit Cell

The Kozeny–Carman equation is a respected relationship used in porous media studies to estimate the permeability of isotropic porous media and thereby predict the pressure drop across it [54,55]. This equation can be expressed as follows:

(A1) $$k=\frac{d_{\sigma }^{2}{\varepsilon_{\beta }}^{3}}{150{\left(1-\varepsilon_{\beta }\right)}^{2}}$$

where ***k*** is the permeability of the porous medium (in square meters), $d_{\sigma }$ is the particle diameter (in meters), and $\varepsilon_{\beta }$ is the porosity of the medium (unit less) representing the fraction of the volume of voids over the total volume.

This equation links the microstructural properties of the porous medium (expressed in terms of particle size and porosity) to its permeability, thereby facilitating the calculation of fluid flow characteristics such as the pressure drop using Darcy’s Law.

Darcy’s Law, in its original form, is usually expressed as follows:

(A2) $$Q=\frac{kA\Delta P_{\beta }}{\mu L}$$

where *Q* is the volume flow rate, *k* is the permeability calculated from Kozney–Carmen equation, $\frac{\Delta P_{\beta }}{L}$ is the pressure drop per unit length, and μ is the dynamic viscosity of the fluid. The calculations for $\frac{\Delta P_{\beta }}{L}$ for all the three experimental cases discussed in Section 4 are presented in Table A1.

**Table A1:** Permeability and pressure drop calculations.

	Case I	Case II	Case III
*k* [m^2^]	1.01 × 10^−9^	3.98 × 10^−10^	8.47 × 10^−10^
$\frac{\Delta P_{\beta }}{L}$ [$\frac{Pa}{m}$]	174.7653	20,182.96	2904.607

## Appendix B. Calculation of Characteristic Time (τ) for Cases I, II, and III Using the Diffusion Equation

The characteristic time in Equation (19) is calculated by utilizing the definition of concentration *c*(*x_k_*,*t_k_*) at any space *x_k_* and time *t_k_* and is explained by the following one-dimensional equation [56]:

(A3) $$C\left(x_{k},t_{k}\right)=C_{in}-\Delta C_{i}({(x}_{k}-x_{i}),{(t}_{k}-t_{i}))$$

where *c_in_* is the initial concentration of the unit cell [Figure 1b] for the porous medium; *t_i_* is the instant of time at which the transport of contaminant occurs for the ith cell, located at $x_{i};$ Δ*c_i_* is the induced or difference in concentration at the point $x_{k}$ at time moment $t_{k}$ with respect to the predecessor single-point pore source located at $x_{i}$ = 0 with time $t_{i}\;$= 0. For the dominant diffusion process, the evolution of the induced concentration (Δ*c_i_*) can be described by the analytic solution of the following one-dimensional diffusion equation:

(A4) $$\frac{\partial C}{\partial t}=D\frac{\partial^{2}C}{\partial x^{2}}$$

Considering the diffusion process with pulse injection of tracer amount M under the boundary condition C(x,t=0)=Mf(x), where f(x) is the delta function described by $\int_{-\infty }^{+\infty }f\left(x\right)=1,$ f(x≠0)=0, the analytic solution for Equation (A4) is obtained [45,52] as follows:

(A5) $$\Delta C_{i}(t,x)=\frac{M}{\sqrt{4\Pi D\left(t\right)}}\exp\left(\frac{-(x^{2})}{4Dt}\right)$$

Substituting Equation (A5) in Equation (A4), the concentration of pore source located at x_k_ for time t_k_ is given as follows:

(A6) $$C\left(x_{k},t_{k}\right)-C_{in}=-\frac{M}{\sqrt{4\Pi D\left(t_{k}-t_{i}\right)}}\;\exp\left(\frac{-((x_{k}-x_{i}{)}^{2})}{4(t_{k}-t_{i})D}\right)$$

The trace amount transported from predecessor pore to the successive point pore is assumed to be identity, i.e., M = 1. At a predetermined initial concentration [$C\left(x_{k},t_{k}\right)=50\;µg/L$] and the initial concentration $C_{in}$ = 100 µg/L, the inhouse MATLAB code written for Equation (A6) is utilized for computing the characteristic time for each of the three cases considered (i.e., Cases I, II, and III) using the parameters in Table A2, respectively:

**Table A2:** The parameter values for each case.

Parameters	Case I	Case II	Case III
*D* (Diffusivity)	6.2 × 10^−10^ m^2^/s	11.6 × 10^−10^ m^2^/s	10^−9^ m^2^/s
$l_{{\tilde{c}}_{\beta }\;}$ (length)	0.75 mm	0.61 mm	0.51 mm

Figure A1 shows the comparison plot for the time taken (represented by dashed color line) to reach the pre-determined induced concentration, for each case, which is referred to as the characteristic time (τ). The characteristic time is evaluated here to estimate whether the present VAM model follows or violates the time constraint given by Equation (19).

### Characteristic Time Calculation for Cases I, II, and III Using the Convection–Diffusion Species Transport Equation

In this section, the characteristic time has been calculated using the convection–diffusion equation within the unit cells for all three cases discussed in Section 4. The convection–diffusion equation, along with the solid-surface adsorption boundary condition, are detailed in Equations (1) and (2). (The flow field needed to solve Equation (1) is computed by solving for Equations (3)–(5) and suitable velocity and pressure conditions on the six faces of the cubical unit cell containing a spherical particle in the middle). Figure A2 illustrates a typical unit cell and some of the applied boundary conditions, providing a visual representation of the geometry used in our simulations.

The simulations were conducted with varying mesh sizes to ensure accuracy and convergence, as presented in Table 7 and Table 10. These tables detail the different mesh sizes used for each case. The choice of mesh size where the numerical results no longer vary with mesh refinement is crucial for accurate simulation. Details about mesh selection has been provided in Section 4.

In these simulations, the characteristic time is defined as the time required for the downstream boundary of the unit cell to reach 50% of the upstream concentration. This definition is significant, as it provides a standardized measure to compare the performance and behavior of different unit cells under various conditions. The downstream boundary’s concentration reaching 50% of the upstream concentration is an indicator of how quickly and efficiently the substance is transported through the unit cell.

Figure A3 shows the results of these simulations, offering a bar-chart view of the characteristic times calculated for each of the three considered cases.
